# Receptor-Associated Prorenin System in the Trabecular Meshwork of Patients with Primary Open-Angle Glaucoma and Neovascular Glaucoma

**DOI:** 10.3390/jcm9082336

**Published:** 2020-07-22

**Authors:** Erdal Tan Ishizuka, Atsuhiro Kanda, Yasuhiro Shinmei, Takeshi Ohguchi, Yoshiaki Tagawa, Keitaro Hase, Taku Yamamoto, Kousuke Noda, Shinki Chin, Susumu Ishida

**Affiliations:** Laboratory of Ocular Cell Biology and Visual Science, Department of Ophthalmology, Faculty of Medicine and Graduate School of Medicine, Hokkaido University, Sapporo, Hokkaido 060-8638, Japan; erdaltan@gmail.com (E.T.I.); yshinmei@med.hokudai.ac.jp (Y.S.); tohguchi@med.hokudai.ac.jp (T.O.); yocchinn0127@med.hokudai.ac.jp (Y.T.); k.hase59@med.hokudai.ac.jp (K.H.); taku.yamamoto@med.hokudai.ac.jp (T.Y.); nodako@med.hokudai.ac.jp (K.N.); schin@med.hokudai.ac.jp (S.C.); ishidasu@med.hokudai.ac.jp (S.I.)

**Keywords:** angiogenesis, angiotensin II type 1 receptor, neovascular glaucoma, primary open-angle glaucoma, prorenin, (pro)renin receptor, receptor-associated prorenin system, renin-angiotensin system, tissue remodeling, trabecular meshwork

## Abstract

The receptor-associated prorenin system (RAPS) is associated with several pathologic conditions, including diabetic retinopathy, age-related macular degeneration, and uveitis. Here, we show the involvement of RAPS in the trabecular meshwork (TM) from patients with primary open-angle glaucoma (POAG) and neovascular glaucoma (NVG) due to proliferative diabetic retinopathy. Anterior chamber (AC) levels of prorenin significantly increased in both POAG and NVG, as did those of angiotensin II in NVG alone, compared to cataract. In surgically excised TM tissues, (pro)renin receptor ((P)RR) and angiotensin II type 1 receptor (AT1R) co-localized with prorenin and angiotensinogen, respectively. In screening for various genes related to glaucoma, prorenin stimulation to human TM cells exclusively upregulated cell junction constituents *connexin 43* and *zona occludens 1*, while downregulating an extracellular matrix-degrading enzyme *tissue plasminogen activator*, all of which were reversed by (P)RR blockade. In contrast, angiotensin II application upregulated a pro-angiogenic factor *placental growth factor* alone, which was abolished by AT1R blockade. Consistently, (P)RR and AT1R co-localized with these corresponding proteins in patient TM tissues. Oxidative stress, a known etiology for glaucoma, induced the expression of *prorenin* and *angiotensinogen* in human TM cells. These data suggest the contribution of RAPS to the molecular pathogenesis of POAG and NVG through TM tissue remodeling and AC angle angiogenesis.

## 1. Introduction

Glaucoma, a neurodegenerative disease with progressive retinal ganglion cell death, is a major cause of irreversible blindness worldwide [1]. Primary open-angle glaucoma (POAG) is the most common form; however, its etiology remains incompletely understood as it appears to be multifactorial [2]. Intraocular pressure (IOP) rise, a proven risk factor for glaucoma, is caused by dysregulation of episcleral venous pressure [3] and increased resistance of anterior chamber (AC) fluid outflow through the trabecular meshwork (TM) located circumferentially at the AC angle, where TM cell junctions [4,5,6] and extracellular matrix (ECM) turnover [7,8,9,10] regulate AC fluid outflow through TM tissue remodeling. Neovascular glaucoma (NVG) is one of the most devastating complications of ischemic retinal disorders including proliferative diabetic retinopathy (PDR). The pathogenesis of NVG is characterized by fibrovascular proliferation at the iris and AC angle, which can ultimately obstruct AC fluid outflow via the TM tissue so as to elevate IOP [11]. Previous reports demonstrated AC fluid profiles of inflammatory, angiogenic, and fibrogenic cytokines in patients with NVG and POAG [10,11,12,13,14,15]; however, the molecular mechanism of glaucoma remains largely elusive.

Renin-angiotensin system (RAS) contributes to the control of systemic homeostasis for blood pressure and water balance, which is commonly known as circulatory (or systemic) RAS. In addition, various organs were shown to express the molecular constituents of RAS, which is termed tissue (or local) RAS. Tissue RAS, acting independently of circulatory RAS, causes inflammation and angiogenesis in the retina and choroid via angiotensin II type 1 receptor (AT1R) signaling [16,17,18,19,20]. Given that tissue RAS was also shown to play facilitatory roles in retinal neuronal dysfunction [21], corneal neovascularization [22], and lacrimal gland fibrosis [23], its ocular involvement covers multiple regions in the eye. As concerns glaucoma, RAS blockade with AT1R blockers achieved IOP reduction in human and monkey glaucoma eyes [24,25] and IOP-independent neuroprotection in rodent glaucoma models [26,27], suggesting a possible etiology of tissue RAS activation and its therapeutic implication in glaucoma [28,29,30]. However, worldwide clinical trials failed to show the adequate efficacy of AT1R blockade for lowering IOP, suggesting the limited contribution of AT1R signaling to the regulation of IOP [31].

Tissue RAS is initiated by prorenin binding to (pro)renin receptor ((P)RR), which exerts renin activity through the conformational change of prorenin (i.e., activated prorenin causing tissue RAS), while simultaneously triggering AT1R-independent signaling pathways in cells expressing (P)RR. This dual bioaction, referred to as “receptor-associated prorenin system” (RAPS), was recently confirmed as the pathogenic etiology of ocular, renal, and cardiac diseases [32,33,34,35,36]. Our clinical sample data also revealed (P)RR involvement in retinal angiogenesis and systemic inflammation in patients with PDR, showing a close association of RAPS with the molecular mechanism of PDR [37,38,39]. Moreover, increasing evidence has suggested RAPS activation in various human eye disorders including age-related macular degeneration [40], idiopathic epiretinal membrane [41], noninfectious uveitis [42], and conjunctival lymphoma [43].

To the best of our knowledge, nothing is known about the involvement of (P)RR signaling in the pathogenesis of glaucoma, although the association of RAS with IOP control was shown to be implicated in the literature [24,25,28,29,30,44]. In this study, using patient surgical samples, we sought to determine the establishment of RAPS in the TM microenvironment and its contribution to the molecular pathogenesis of POAG and NVG.

## 2. Methods

### 2.1. Clinical Characteristics of Patients

The clinical characteristics of patients are listed in Table 1. We studied 11 eyes of 11 non-diabetic cases of POAG (5 males and 6 females, average age = 65.6 ± 3.8 years), 11 eyes of 11 cases of NVG secondary to PDR (6 males and 5 females, average age = 62.6 ± 2.5 years), and 7 eyes of 7 non-diabetic cases of age-related cataract (4 males and 3 females, average age = 70.7 ± 2.0 years) that served as age- and sex-matched controls for comparison of AC fluid data. Most of the patients enrolled here had age- and/or lifestyle-related systemic diseases. The mean values of pre-operative IOP on anti-glaucoma medications were 18.2 ± 1.6 mmHg and 31.9 ± 2.8 mmHg in eyes with POAG and NVG, respectively, both of which were higher than the pre-operative IOP value of 14.4 ± 0.9 mmHg in eyes with cataract. The study was performed according to the tenets of the Declaration of Helsinki after it was approved by the institutional review board of Hokkaido University Hospital (#011-0204). All patients gave written informed consent following our explanation of the purpose and procedures of the study.

### 2.2. Patient Surgical Samples

AC fluids were collected from 7 patients with POAG (4 males and 3 females, average age = 62.3 ± 4.4 years), 6 patients with NVG (4 males and 2 females, average age = 64.5 ± 3.1 years), and 7 control patients with cataract. AC fluids were aspirated by limbal paracentesis at the start of surgery. Undiluted AC fluids were frozen immediately and stored at -80 °C until enzyme-linked immunosorbent assay (ELISA). TM tissues were collected from 4 patients with POAG and 5 patients with NVG. TM tissues were surgically excised during trabeculectomy, and used for immunofluorescence, immunohistochemical, and gene expression analyses.

### 2.3. Cell Culture and Chemicals

TM cells, isolated from a glaucoma patient (a kind gift of Novartis Institutes for BioMedical Research, Cambridge, MA, USA), were incubated in D-MEM supplemented with 10% fetal bovine serum [45]. To replicate the binding site of prorenin to (P)RR (i.e., the handle region of prorenin), decoy peptides NH_2_-RIFLKRMPSI-COOH were synthesized as human (P)RR blocker (PRRB) [37] (GeneDesign, Osaka, Japan). After serum deprivation, TM cells at sub-confluency were pretreated with PRRB (1 μM) or AT1R blocker valsartan (10 μM) (Sigma-Aldrich, St. Louis, MO, USA) for 1 h, followed by stimulation with prorenin (10 nM) or angiotensin (Ang) II (1 μM). Cells were cultured for 24 h and processed for analysis of mRNA expression levels. TM cells were also treated with hydrogen peroxide (H_2_O_2_) at 30, 60, and 90 μM for 24 h, and analyzed for the gene expression of RAPS components [*(P)RR*, *REN* (prorenin), *AGT* (angiotensinogen), *AT1R*, and *ACE* (angiotensin-converting enzyme)]. Phosphate-buffered saline was used as a vehicle control.

### 2.4. ELISA

The protein levels of prorenin and Ang II in patient AC fluids were measured with ELISA kits for human prorenin (Abcam, Cambridge, MA, USA) and Ang II (Bertin Pharma, Montigny Le Bretonneux, France), according to the manufacturers’ instructions. The optical density was determined with the Sunrise microplate reader (TECAN, Männedorf, Switzerland).

### 2.5. Immunofluorescence Microscopy

TM tissue samples surgically excised from patients were embedded in paraffin after fixation in 4% paraformaldehyde. Paraffin sections of TM tissues were dewaxed, rehydrated, and rinsed in PBS. As a pretreatment, microwave-based antigen retrieval was carried out in 10 mM citrate buffer (pH 6.0). Sections were probed with the following primary antibodies: rabbit anti-(P)RR (Sigma-Aldrich), mouse anti-prorenin (Abcam), rabbit anti-AT1R, goat anti-AGT, goat anti-tissue plasminogen activator (t-PA), goat anti-placental growth factor (PlGF) (Santa Cruz Biotechnology, Santa Cruz, CA, USA), mouse anti-connexin (CX)43, and rat anti-zona occludens (ZO)-1 (Millipore, Temecula, CA, USA) antibodies. Normal mouse, goat, and rabbit IgGs were used as negative control antibodies. Secondary antibodies for fluorescent detection were labeled with Alexa Fluor 488 and Alexa Fluor 546 (Thermo Fisher Scientific, Waltham, MA, USA). Sections were visualized under the BIOREVO BZ-9000 fluorescence microscope (Keyence, Osaka, Japan).

### 2.6. Immunohistochemistry

The slides were pre-incubated with normal goat serum to block non-specific binding, followed by peroxidase block solution to block endogenous peroxidase activity. Sections were probed with rabbit anti-(P)RR (Sigma-Aldrich) and rabbit anti-AT1R (Santa Cruz Biotechnology) primary antibodies. Color was developed using the Envision HRP kit (DAKO, Carpinteria, CA, USA). Normal rabbit IgG was used as a negative control antibody. Sections were analyzed using the BIOREVO microscope (Keyence).

### 2.7. Reverse Transcription-PCR (RT-PCR) and Real-Time Quantitative PCR (qPCR) Analyses

Total RNA isolation was performed from cultured TM cells using TRIzol (Thermo Fisher Scientific) and from excised TM tissues using the NucleoSpin totalRNA FFPE XS kit (Macherey-Nagel, Düren, Germany), followed by reverse transcription with GoScript Reverse Transcriptase (Promega, Madison, WI, USA), as described previously [37]. GoTaq qPCR Master Mix (Promega) and StepOnePlus System (Thermo Fisher Scientific) were used for the gene expression assay. To normalize gene expression data, human *B2M* (β2-microglobulin) gene was used as an internal control. Gene expression levels were calculated using the ddCt method. The primers used in this study are listed in Table S1.

### 2.8. Statistical Analyses

All the results are presented as the mean ± SEM (standard error of the mean). The Mann–Whitney U test was used for pairwise comparison between groups, and the Kruskal–Wallis test for multiple comparison. Differences were considered as statistically significant at *p* values < 0.05.

## 3. Results

### 3.1. Elevation of Prorenin and Ang II Levels in AC Fluids from POAG and NVG Patients

**Figure 1:**
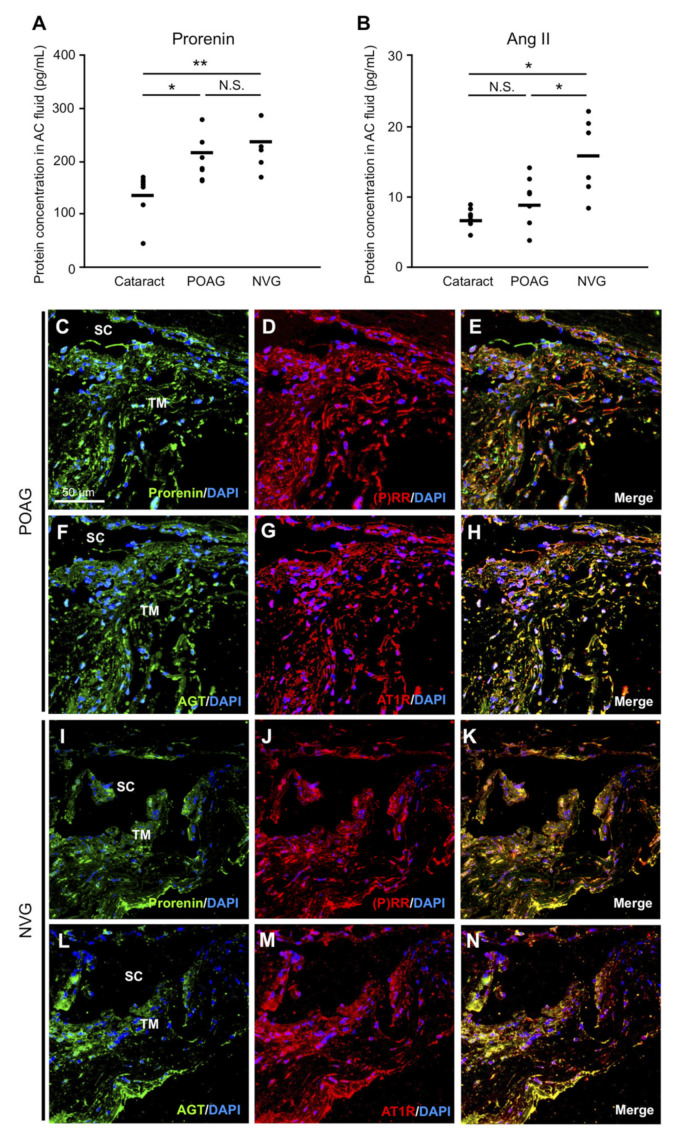
Prorenin and Ang II levels in AC fluids and ligand-receptor co-localization in TM tissues from POAG and NVG patients. (**A**) Prorenin protein levels in eyes with cataract, POAG, and NVG. (**B**) Ang II protein levels in eyes with cataract, POAG, and NVG. Black dots indicate individual samples, and bars show the mean. * *p* < 0.05, ** *p* < 0.01, N.S., not significant. (**C**–**N**) Double labeling of prorenin (green) and (P)RR (red) with DAPI (blue) (**C**–**E**,** I**–**K**) and of AGT (green) and AT1R (red) with DAPI (blue) (**F**–**H**,** L**–**N**) in TM tissues from POAG (**C**–**H**) and NVG (**I**–**N**) eyes. Scale bar = 50 μm. SC, Schlemm’s canal. ((P)RR: (pro)renin receptor, AGT: angiotensinogen, AT1R: angiotensin II type 1 receptor.

To investigate the intraocular production of prorenin and Ang II in glaucoma patients, we performed ELISA to measure these protein levels in AC fluids collected from POAG and NVG eyes, together with control eyes with cataract. Prorenin protein levels in eyes with POAG (203.3 ± 15.79 pg/mL, *p* < 0.05) and NVG (232.0 ± 19.24 pg/mL, *p* < 0.01) were significantly higher than those with cataract (138.2 ± 16.79 pg/mL) (Figure 1A). Ang II levels significantly increased in eyes with NVG (15.7 ± 2.27 pg/mL, *p* < 0.05) compared with cataract (7.06 ± 0.54 pg/mL) and POAG (9.49 ± 1.34 pg/mL) (Figure 1B). There was a non-significant trend for Ang II elevation in eyes with POAG compared to cataract (*p* = 0.13). With regard to receptors for prorenin and Ang II, we performed immunohistochemical analyses. Both (P)RR and AT1R were immunopositive in TM cells of TM tissues excised from POAG and NVG eyes (Figure S1A–F), in accordance with the presence of their ligands in AC fluids.

### 3.2. RAPS Ligand–Receptor Co-localization in TM Tissues from POAG and NVG Patients

To further examine RAPS ligand-receptor interaction in TM tissues, we performed immunofluorescence analyses. Double-staining results showed co-localization of (P)RR with prorenin (Figure 1C–E, I–K) and AT1R with AGT (Figure 1F–H, L–N) in TM tissues from POAG (Figure 1C–H) and NVG (Figure 1I–N) eyes. In agreement with the ligand–receptor co-localization, the RT-PCR results showed the overall expression of RAPS component genes (*(P)RR*, *REN*, *AGT*, *AT1R*, and *ACE*) in TM tissues from POAG and NVG eyes (Figure S1G). Normal isotype IgGs showed no immunostaining (Figure S2A–F). These findings suggested the potential contribution of prorenin–(P)RR and Ang II–AT1R axes to the pathogenesis of glaucoma in the TM microenvironment.

### 3.3. Selection of Target Genes Related to the Pathogenesis of POAG and NVG

**Figure 2:**
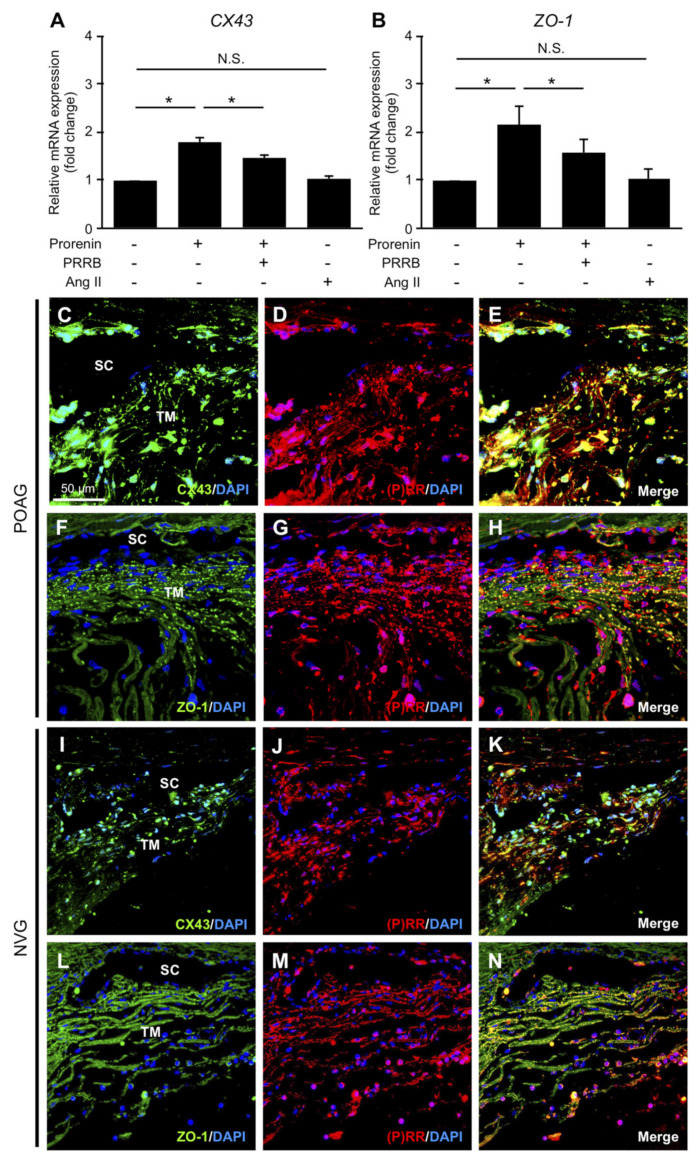
Upregulation of *CX43* and *ZO-1* via prorenin–(P)RR interaction in TM cells and their production in TM tissues from POAG and NVG patients. (**A**,**B**) Relative mRNA expression levels of *CX43* (**A**) and *ZO-1* (**B**) in prorenin- or Ang II-stimulated TM cells with or without PRRB. *n* = 5. * *p* < 0.05. N.S., not significant. (**C**–**N**) Double labeling of CX43 (green) and (P)RR (red) with DAPI (blue) (**C**–**E**, **I**–**K**) and of ZO-1 (green) and (P)RR (red) with DAPI (blue) (**F**–**H**, **L**–**N**) in TM tissues from POAG (**C**–**H**) and NVG (**I**–**N**) eyes. Scale bar = 50 μm. SC, Schlemm’s canal.

**Figure 3:**
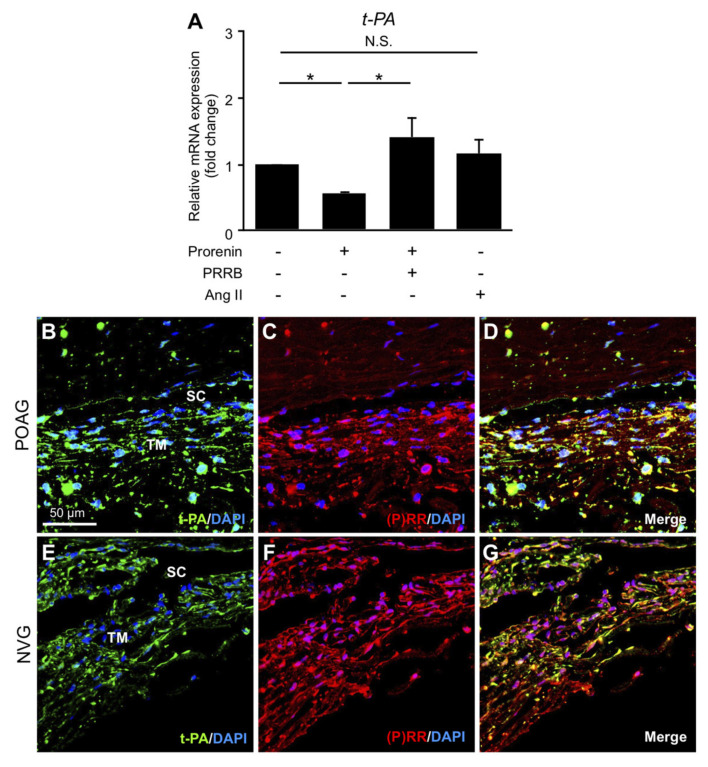
Downregulation of *t-PA* via prorenin–(P)RR interaction in TM cells and its production in TM tissues from POAG and NVG patients. (**A**) Relative mRNA expression levels of *t-PA* in prorenin- or Ang II-stimulated TM cells with or without PRRB. *n* = 6. * *p* < 0.05. N.S., not significant. (**B**–**G**) Double labeling of t-PA (green) and (P)RR (red) with DAPI (blue) in TM tissues from POAG (**B**–**D**) and NVG (**E**–**G**) eyes. Scale bar = 50 μm. SC, Schlemm’s canal.

**Figure 4:**
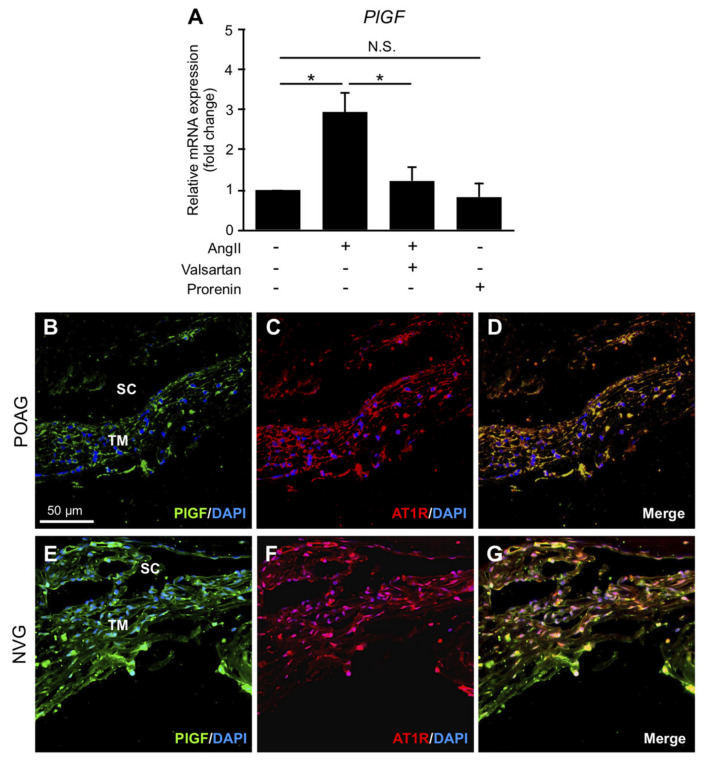
Upregulation of *PlGF* via Ang II–AT1R interaction in TM cells and its production in TM tissues from POAG and NVG patients. (**A**) Relative mRNA expression levels of *PlGF* in Ang II- or prorenin-stimulated TM cells with or without AT1R blocker valsartan. *n* = 6. * *p* < 0.05. N.S., not significant. (**B**–**G**) Double labeling of PlGF (green) and AT1R (red) with DAPI (blue) in TM tissues from POAG (**B**–**D**) and NVG (**E**–**G**) eyes. Scale bar = 50 μm. SC, Schlemm’s canal. *PlGF:* goat anti-placental growth factor

A number of studies have reported that various molecules are involved in the pathogenesis of glaucoma [4,5,6,7,8,9,10,11,12,13,14,15]. On the basis of our literature search, we selected representative molecules (Table S2) related to angiogenesis (e.g., PlGF and vascular endothelial growth factor A) [11,15], cell junction (e.g., CX43 alternatively called as gap junction alpha-1 protein (GJA1) and ZO-1 alternatively called as tight junction protein 1 (TJP1)) [4,5,6], ECM turnover (e.g., matrix metalloproteinases and t-PA) [7,8,9], fibrosis (e.g., transforming growth factor-β2) [10,14], inflammation (e.g., C–C motif chemokine ligand 2 alternatively called as monocyte chemotactic protein 1) [12,13], and oxidative stress (e.g., uncoupling protein 2) [5]. To investigate the role of RAPS in the molecular pathogenesis of glaucoma, we screened these various genes via real-time qPCR in human TM cell culture stimulated with either prorenin or Ang II. Of these listed here, we consequently determined in the following experiments (Figure 2, Figure 3 and Figure 4) molecules characterized by altered gene expression in the in vitro screening as well as protein co-localization with the corresponding receptors in patient TM tissues.

### 3.4. Upregulation of CX43 and ZO-1 via Prorenin–(P)RR Interaction in TM Cells and Their Production in TM Tissues from POAG and NVG Patients

CX43 and ZO-1, gap and tight junction proteins expressed in TM cells, were suggested to contribute to the resistance of AC fluid outflow [4,5,6]. Expression levels of *CX43* and *ZO-1* significantly increased in TM cells stimulated with prorenin compared to vehicle (*CX43,* fold change = 1.78; *ZO-1,* fold change = 2.17, *p* < 0.05), both of which were repressed by pretreatment with PRRB (*CX43*, fold change = 1.47; *ZO-1*, fold change = 1.57, *p* < 0.05) (Figure 2A,B). In contrast, Ang II application did not change their expression levels (*CX43*, fold change = 1.04; *ZO-1*, fold change = 1.02). Immunofluorescence analyses demonstrated co-localization of CX43 and ZO-1 with (P)RR in TM tissues from POAG (Figure 2C–H) and NVG (Figure 2I–N) eyes, supporting the upregulation of *CX43* and *ZO-1* expression via (P)RR signaling pathway in TM cells. These results indicated the possible contribution of prorenin–(P)RR axis to TM tissue remodeling via augmented junctional resistance in POAG and NVG eyes.

### 3.5. Downregulation of t-PA via Prorenin–(P)RR Interaction in TM Cells and Its Production in TM Tissues from POAG and NVG Patients

The regulation of AC fluid outflow was shown to depend on t-PA-mediated ECM turnover in the TM tissue [7,8,9,10]. Expression levels of *t-PA* significantly decreased in TM cells stimulated with prorenin compared to vehicle (fold change = 0.56, *p* < 0.05), which was repressed by pretreatment with PRRB (fold change = 1.41, *p* < 0.05) (Figure 3A). In contrast, Ang II application did not change its expression levels (fold change = 1.17, *p* > 0.05). Immunofluorescence analyses demonstrated co-localization of t-PA with (P)RR in TM tissues from POAG (Figure 3B–D) and NVG (Figure 3E–G) eyes, supporting the downregulation of *t-PA* expression via (P)RR signaling pathway in TM cells. These results indicated the possible contribution of prorenin–(P)RR axis to TM tissue remodeling via delayed ECM turnover in POAG and NVG eyes.

### 3.6. Upregulation of PlGF via Ang II–AT1R Interaction in TM Cells and Its Production in TM Tissues from POAG and NVG Patients

PlGF, a pro-angiogenic factor elevated in eyes with NVG, was theorized to play a role in the regulation of AC angle angiogenesis [15]. Expression levels of *PlGF* significantly increased in TM cells stimulated with Ang II compared to vehicle (fold change = 2.93, *p* < 0.05), which was repressed by pretreatment with AT1R blocker valsartan (fold change = 1.24, *p* < 0.05) (Figure 4A). In contrast, prorenin application did not change its expression levels (fold change = 0.82, *p* > 0.05). Immunofluorescence analyses demonstrated co-localization of PlGF with AT1R in TM tissues from POAG (Figure 4B–D) and NVG (Figure 4E–G) eyes, supporting the upregulation *PlGF* expression via AT1R signaling pathway in TM cells. Notably, the faint PlGF expression in TM tissues from POAG compared to NVG eyes (Figure 4B,E) was consistent with the significant elevation of Ang II in NVG but not POAG eyes (Figure 1B). These results indicated the possible involvement of Ang II–AT1R axis in AC angle angiogenesis in NVG eyes.

### 3.7. Upregulation of RAPS Ligands but Not Receptors in TM Cells Exposed to Oxidative Stress

**Figure 5:**
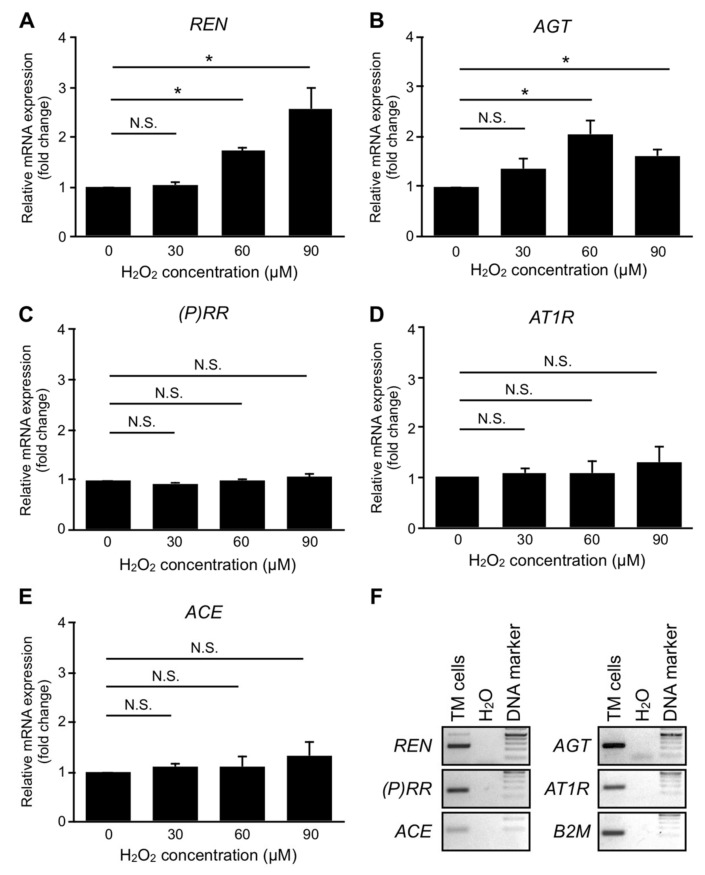
Upregulation of RAPS ligands but not receptors in TM cells exposed to oxidative stress. (**A**–**E**) Relative mRNA expression levels of RAPS component genes *REN*, *AGT*, *(P)RR*, *AT1R*, and *ACE* in TM cells with H_2_O_2_ exposure. *n* = 6. * *p* < 0.05. N.S., not significant. (**F**) Constitutive expression of RAPS component genes in TM cells at steady-state levels. *B2M* was used as an internal control. Composite images from multiple DNA agarose gel electrophoresis. RAPS: Upregulation of receptor-associated prorenin system, *ACE:* angiotensin-converting enzyme.

Oxidative stress was proven to increase in the AC fluid and the TM tissue of patients with glaucoma, and has been regarded as a significant factor linked with IOP elevation [46,47,48,49,50]. We next investigated the impact of oxidative stress on the in vitro expression of RAPS component genes. Compared to vehicle, H_2_O_2_ exposure significantly upregulated the expression levels of *REN* (60 μM, fold change = 1.75; 90 μM, fold change = 2.56, *p* < 0.05) (Figure 5A) and *AGT* (60 μM, fold change = 2.03; 90 μM, fold change = 1.59, *p* < 0.05) (Figure 5B) in human TM cells. No significant changes were detected in the expression levels of *(P)RR*, *AT1R*, or *ACE* (Figure 5C–E), suggesting the ligand-based activation of RAPS in the TM tissue under oxidative stress. In line with the real-time qPCR data, RT-PCR results showed the concomitant expression of RAPS ligand–receptor pairs in TM cells (Figure 5F), supporting the molecular mechanisms of TM RAPS via autocrine regulation (Figure 2, Figure 3 and Figure 4). Notably, the faint *ACE* expression was consistent with the comparably low Ang II levels in POAG eyes (Figure 1B).

## 4. Discussion

**Figure 6:**
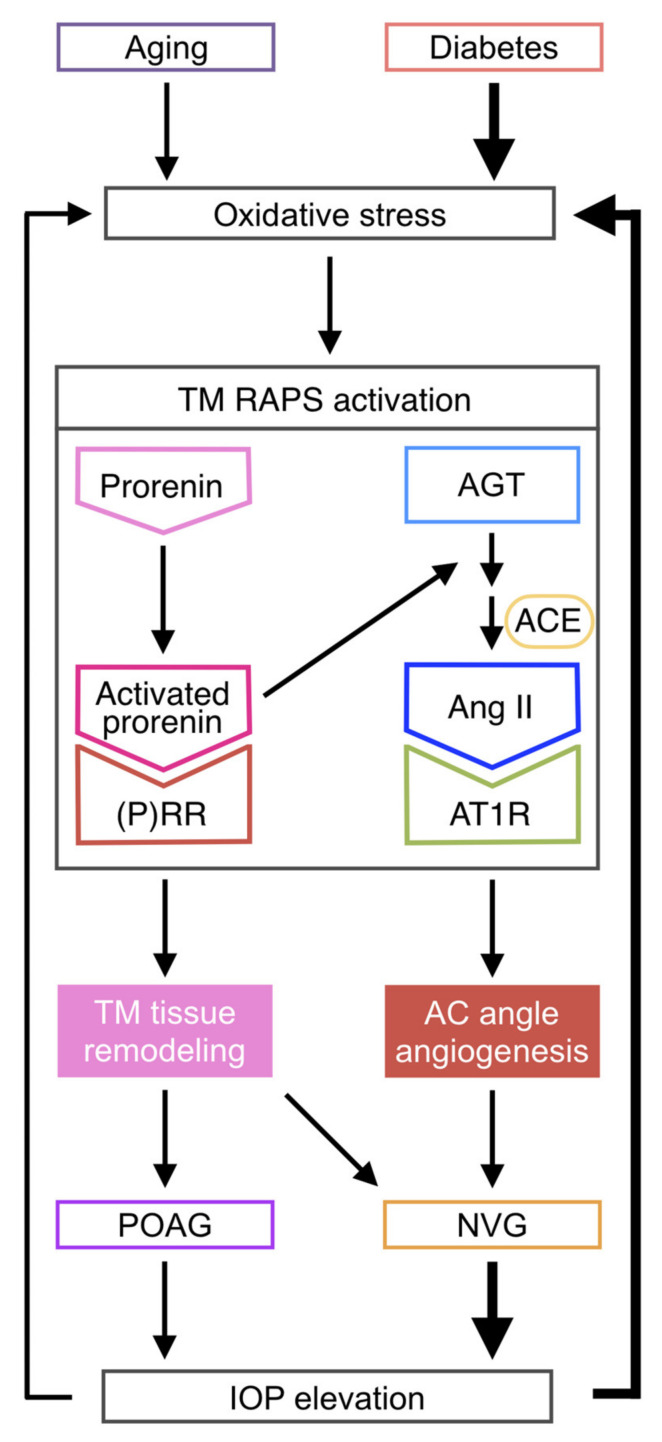
A schema showing the involvement of TM RAPS in the pathogenesis of POAG and NVG via TM tissue remodeling and AC angle angiogenesis. Glaucoma-associated activation of TM RAPS exerts prorenin–(P)RR and Ang II–AT1R axes for TM tissue remodeling and AC angle angiogenesis, respectively. TM RAPS mediates the vicious cycle between oxidative stress and IOP elevation more likely to be enhanced in NVG compared to POAG [48].

This study provided the first evidence for the crucial roles of RAPS in the molecular pathogenesis of glaucoma, using surgical samples collected from patients with POAG and NVG. Elevated AC levels of prorenin were detected in both POAG and NVG, together with those of Ang II in NVG alone (Figure 1). In patient TM tissues, (P)RR and AT1R co-localized with prorenin and AGT, respectively (Figure 1). In screening for various glaucoma-related genes, prorenin stimulation to TM cells exclusively upregulated cell junction genes *CX43* and *ZO-1* (Figure 2), while downregulating an ECM regulatory gene *t-PA* (Figure 3), all of which were reversed by the (P)RR blockade (Figure 2 and Figure 3). In contrast, Ang II application upregulated a pro-angiogenic gene *PlGF* alone, which was abolished by the AT1R blockade (Figure 4). Consistently, (P)RR and AT1R co-localized with these corresponding proteins in patient TM tissues, supporting the (P)RR- and AT1R-mediated regulation of molecules related to TM tissue remodeling and AC angle angiogenesis, respectively (Figure 2, Figure 3 and Figure 4). These results, based on patient samples, support TM RAPS as a potential molecular factor in the pathogenesis of POAG and NVG. (Figure 6).

Moreover, human TM cells exposed to oxidative stress, a known etiology for glaucoma [46,47,48,49,50], selectively upregulated the RAPS ligand genes *REN* and *AGT*, while all the major RAPS components including their receptors were expressed at steady-state levels (Figure 5). Given that oxidative stress is the central etiology of aging processes associated with age-related cataract as well as POAG [50], these novel observations are compatible with the presence of prorenin and Ang II in the AC fluid of patients with age-related cataract and POAG (Figure 1). Since IOP rise was shown to increase AC levels of oxidative stress in experimental glaucoma [47], the cause–effect relation between oxidative stress and IOP rise is theorized to be reciprocal, thus forming a vicious cycle. In diabetic patients, hyperglycemia is a predisposing factor for mitochondrial oxidative stress [51], while elevated Ang II in NVG due to PDR (Figure 1) would reasonably lead to cytoplasmic oxidative stress via AT1R-nicotinamide adenine dinucleotide phosphate, reduced form (NADPH) oxidase axis [52]. Indeed, AC levels of oxidative stress were reported to be augmented in NVG compared to POAG [48], in agreement with pronounced IOP rise in NVG compared to POAG (31.9 ± 2.8 mmHg vs. 18.2 ± 1.6 mmHg in this case series). Therefore, the vicious cycle between oxidative stress and IOP rise is more likely to be enhanced in NVG harboring Ang II–AT1R as well as prorenin–(P)RR pathways (Figure 6).

The critical bioaction of (P)RR bound with prorenin lies in its own signal transduction in various cell species, on top of the exertion of renin enzymatic activity by conformationally altered prorenin (i.e., activated prorenin) to initiate tissue RAS activation causing AT1R signaling [32,33,34,35,36]. In the rat model of diabetic nephropathy, the progression of glomerulosclerosis was shown to largely stem from (P)RR signaling, together with the limited contribution of tissue RAS [36]. In the mouse model of diabetic retinopathy, leukocyte adhesion to the retinal vasculature was revealed to be governed independently by both AT1R and (P)RR [17,32]. Molecules induced via (P)RR downstream pathway and their cellular sources were reported to include monocyte chemotactic protein 1 in brain capillary endothelial cells [34], vascular endothelial growth factor A in macrophages [34] and retinal microvascular endothelial cells [37], transforming growth factor-β1 in retinal pigment epithelial cells [40], and fibroblast growth factor 2 in Müller glial cells [41]. (P)RR signaling would thus lead to various pathologic conditions such as inflammation, angiogenesis, and fibrosis, depending on different cellular reactions in diabetic retinopathy [37], age-related macular degeneration [40], and idiopathic epiretinal membrane [41].

The present study demonstrated, for the first time, TM tissue remodeling as another pathologic condition mediated via (P)RR signaling. Because prorenin increased in both POAG and NVG (Figure 1), this biological event may be part of a common pathogenic mechanism for IOP control. TM tissue remodeling is thought to mitigate AC fluid outflow facility basically through TM cell junctional resistance [4,5,6] and delayed ECM turnover [7,8,9,10]. In relation with these two microenvironmental changes, junctional constituents CX43 and ZO-1 (Figure 2) and an ECM-degrading enzyme t-PA (Figure 3) were suggested as significant molecular contributors to (P)RR-mediated TM tissue remodeling. Theoretically, the currently observed augmentation of the junctional components and reduction of the ECM-digesting mediator would elevate TM tissue resistance against AC fluid outflow. Indeed, *ZO-1* knockdown via small interfering RNA in vitro [4] and *t-PA* overexpression via adenoviral vector in vivo [8] were both shown to alleviate steroid-induced outflow resistance, supporting our results showing the pathogenic link between TM RAPS activation and IOP elevation.

This study is the first to show TM cells as a cellular source for AT1R-mediated production of PlGF (Figure 4), which was recently shown to be elevated in the AC fluid of patients with NVG due to PDR [15]. In agreement with the significant elevation of Ang II in NVG but not POAG (Figure 1), the pro-angiogenic factor PlGF involvement would reasonably be considered as a characteristic molecular mechanism specific for NVG. This would be attributable in part to a potential difference between POAG and NVG in AC levels of ACE and Ang II, both of which were previously shown to increase in the vitreous of eyes with PDR [53,54]. Moreover, vascular endothelial cells were reported to induce PlGF expression via Ang II–AT1R signaling [55], suggesting new vessels on the iris and AC angle as the additional cellular source of PlGF in eyes with NVG. However, PlGF weakly co-localized with AT1R in the TM tissue of eyes with POAG (Figure 4), consistent with the unelevated levels of Ang II in the AC fluid (Figure 1). Besides its pro-angiogenic action, the physiological role of baseline PlGF expression in the TM tissue is now under investigation in our laboratory (manuscript in preparation).

The limitations of the current study are as follows: First, the number of patients included in this study was small. Second, normal TM tissues were not compared due to a legislative difficulty in gaining cadaveric eyes. Third, the degree of oxidative stress in our patient samples was not evaluated. Finally, other stimuli in the upstream of RAPS were not targeted for investigation. These additional examinations would be valuable to further verify our hypothesis in the future.

## 5. Conclusions

The TM microenvironment, per se, was suggested to be fully equipped with the major RAPS components, and the etiologic link between oxidative stress and ocular hypertension would likely be mediated by TM RAPS. Our data support the potential role of TM RAPS in the molecular pathogenesis of POAG and NVG via TM tissue remodeling and AC angle angiogenesis, which were promoted by prorenin–(P)RR and Ang II–AT1R axes, respectively. These results, with patient samples, implicated the potential efficacy of blocking (P)RR as a novel therapeutic strategy for the management of glaucoma.

## Figures and Tables

**Table 1:** Clinical characteristics of patients.

Diagnosis	Age	Sex	Systemic Complications	Pre-Operative IOP (mmHg)	Surgical Samples	Purpose of Use
POAG	80	M	Myocardial infarction	17	AC fluid	ELISA
	75	F	None	27		
	50	F	Internal carotid aneurysm	15		
	50	M	DL	19		
	57	F	None	21		
	64	M	None	17		
	60	M	None	16		
	85	F	HT	14	TM tissue	IF, IHC, RT-PCR
	64	F	None	28		
	80	F	HT	10		
	57	M	HT	16		
NVG secondary to PDR	76	M	DM	40	AC fluid	ELISA
	55	F	DM, DL, HT	46		
	66	F	DM	29		
	68	M	DM	34		
	57	M	DM, Maxillary sinusitis	38		
	65	M	DM, HT	35		
	61	F	DM, DN, HT	29	TM tissue	IF, IHC, RT-PCR
	44	M	DM, HT	21		
	65	F	DM, Megaloblastic anemia	40		
	64	F	DM, DN, HT	21		
	67	M	DM, DN, HT	18		
Cataract	78	F	HT	13	AC fluid	ELISA
	64	F	DL, HT	13		
	73	M	DL	14		
	69	M	HT	19		
	76	M	DL	15		
	70	M	DL	15		
	65	F	DL	12		

AC, anterior chamber; DL, dyslipidemia; DM, diabetes mellitus; DN, diabetic nephropathy; ELISA, enzyme-linked immunosorbent assay; HT, hypertension; IF, immunofluorescence; IHC, immunohistochemistry; IOP, intraocular pressure; NVG, neovascular glaucoma; PDR, proliferative diabetic retinopathy; POAG, primary open-angle glaucoma; RT-PCR, reverse transcription-PCR; TM, trabecular meshwork.

## Supplementary Materials

The following are available online at https://www.mdpi.com/2077-0383/9/8/2336/s1, Figure S1: Localization and expression of RAPS components in TM tissues from POAG and NVG patients. Figure S2: Staining of isotype controls in TM tissues from POAG and NVG patients. Table S1: Primer sequences used in RT-PCR and real-time qPCR. Table S2: Target genes related to the pathogenesis of POAG and NVG.
