# Supplementary material for: Receptor-Associated Prorenin System in the Trabecular Meshwork of Patients with Primary Open-Angle Glaucoma and Neovascular Glaucoma
Source: J Clin Med. 2020 Jul 22;9(8):2336. doi: 10.3390/jcm9082336 (PMC7465309; doi:10.3390/jcm9082336)
Supplement: Supplementary file 1 [file jcm-09-02336-s001.pdf]

Supplementary Figure S1

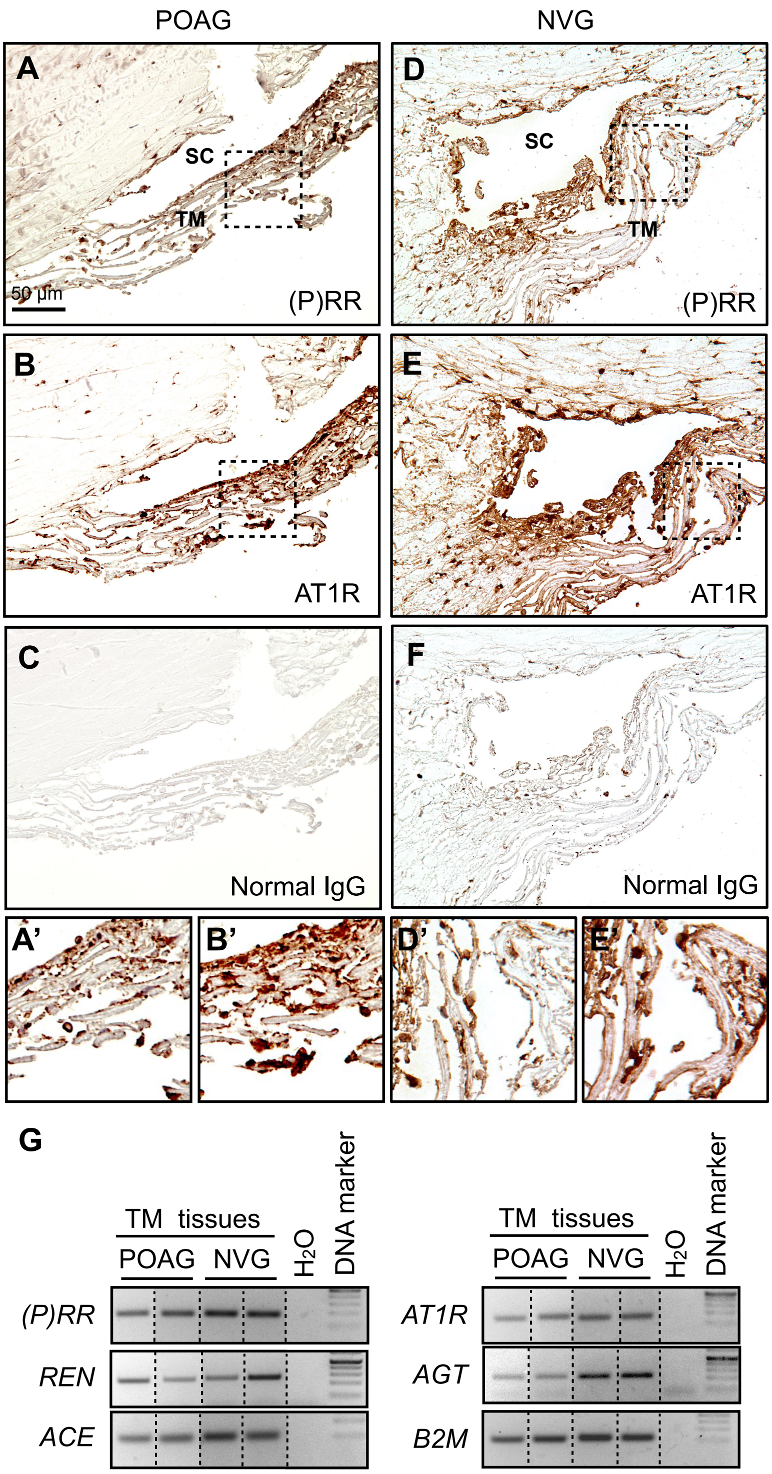

Figure S1. Localization and expression of RAPS components in TM tissues from POAG and NVG patients. (**A-F**) Immunohistochemical staining of (P)RR (**A, D**) and AT1R (**B, E**) in TM tissues from eyes with POAG (**A-C**) and NVG (**D-F**). (**C, F**) No signal was observed with control IgG. (**A', B', D', E'**) Magnified view in the corresponding insets. Scale bar = 50 µm. SC, Schlemm's canal. (**G**) Qualitative expression of RAPS component genes in TM tissues from POAG and NVG eyes. *B2M* was used as an internal control. Representative composite images from multiple DNA agarose gel electrophoresis.

Supplementary Figure S2

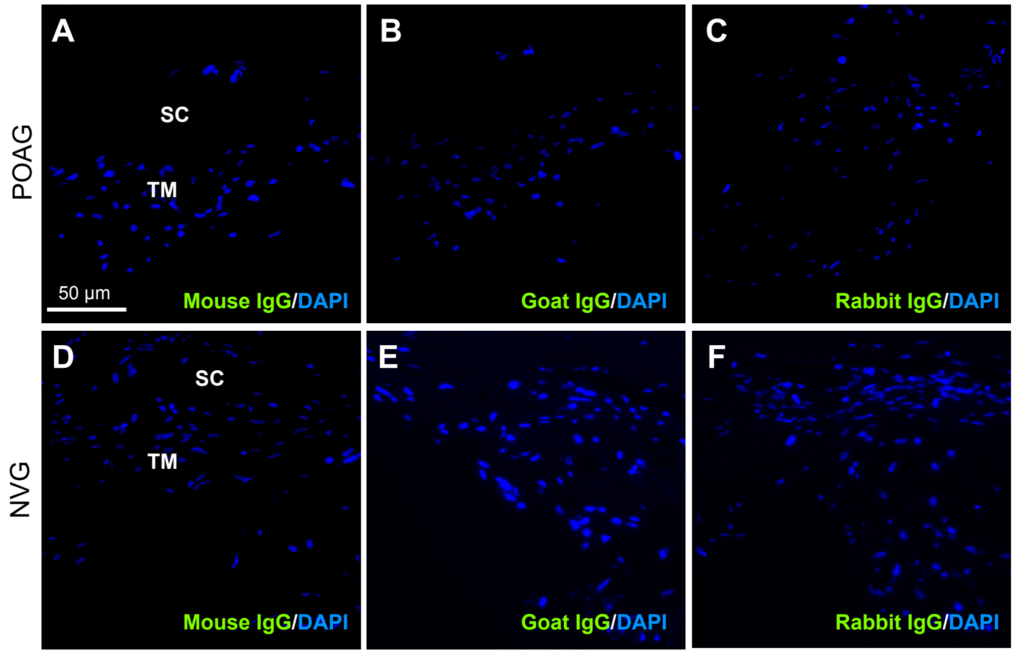

Figure S2. Staining of isotype controls in TM tissues from POAG and NVG patients. Immunofluorescence staining of normal mouse IgG (**A, D**), goat IgG (**B, E**), and rabbit IgG (**C, F**) in TM tissues from eyes with POAG (**A-C**) and NVG (**D-F**). Scale bar = 50 μm. SC, Schlemm's canal.

Supplementary Table S1. Primer sequences used in RT-PCR and real-time qPCR

(P)RR	forward 5’-AGG CAG TGT CAT TTC GTA CC-3’
	reverse 5’-GCC TTC CCT ACC ATA TAC ACT C-3’
REN	forward 5’-GTG TCT GTG GGG TCA TCC ACC TTG-3’
	reverse 5’-GGA TTC CTG AAA TAC ATA GTC CGT-3’
AT1R	forward 5’-AGG GCA GTA AAG TTT TCG TG-3’
	reverse 5’-CGG GCA TTG TTT TGG CAG TG-3’
AGT	forward 5’-CTG CAA GGA TCT TAT GAC CTG C-3’
	reverse 5’-TAC ACA GCA AAC AGG AAT GGG C-3’
ACE	forward 5’-CCG AAA TAC GTG GAA CTC ATC AA-3’
	reverse 5’-CAC GAG TCC CCT GCA TCT ACA-3’
CX43	forward 5’-AGG TCT GAG TGC CTG AAC TTG-3’
	reverse 5’-TTG CCT GGG CAC CAC TCT TTT-3’
ZO-1	forward 5’-ACC AGA AAT ACC TGA CGG TGC-3’
	reverse 5’-CGT TAC CCA CAG CTT CCT CTT-3’
t-PA	forward 5’-CAG AAG CAA CCG GGT GGA ATA-3’
	reverse 5’-CGC TGC AAC TTT TGA CAG GC-3’
PlGF	forward 5’-TCA CCA TGC AGC TCC TAA AGA-3’
	reverse 5’-GTG GCA GTC TGT GGG TCT CT-3’
B2M	forward 5’-GAG TAT GCC TGC CGT GTG AA-3’
	reverse 5’-GCG GCA TCT TCA AAC CTC CA-3’

Supplementary Table S2. Target genes related to the pathogenesis of POAG and NVG

Function	Target Genes	
	Gene symbol	Protein name
Angiogenesis	*EGF*	Epidermal growth factor
	NRP1	Neuropilin 1
	PGF	Placental growth factor (PlGF)
	VEGFA	Vascular endothelial growth factor A
	VEGFR1	Vascular endothelial growth factor receptor 1
	VEGFR2	Vascular endothelial growth factor receptor 2
Cell junction	*GJA1*	Connexin 43 (CX43)
	TJP1	Zona occludens 1 (ZO-1)
ECM turnover	*BSG*	Basigin
	MMP2	Matrix metalloproteinase 2
	MMP3	Matrix metalloproteinase 3
	MMP9	Matrix metalloproteinase 9
	MMP14	Matrix metalloproteinase 14
	PLAT	Tissue plasminogen activator (t-PA)
	TGM2	Transglutaminase 2
	TIMP1	Tissue inhibitor of metalloproteinase 1
Fibrosis	*CDKN1A*	Cyclin dependent kinase inhibitor 1A
	FGF2	Fibroblast growth factor 2
	TGFB2	Transforming growth factor-β2
Inflammation	*CCL2*	C-C motif chemokine ligand 2/ Monocyte chemotactic protein 1
	ICAM1	Intercellular adhesion molecule 1
Oxidative stress	*UCP2*	Uncoupling protein 2
